# Editorial: Cardio-Protection and Heart Repair: New Drugs, Targets and Approaches

**DOI:** 10.3389/fphar.2021.807645

**Published:** 2021-11-29

**Authors:** Naufal Zagidullin, Benzhi Cai, Hua Zhu

**Affiliations:** ^1^ Department of Internal Diseases, Bashkir State Medical University, Ufa, Russia; ^2^ Department of Pharmacy at The Second Affiliated Hospital, Harbin Medical University, Harbin, China; ^3^ Department of Pharmacology (The Key Laboratory of Cardiovascular Medicine Research, Ministry of Education) at College of Pharmacy, Harbin Medical University, Harbin, China; ^4^ Department of Surgery, The Ohio State University Wexner Medical Center, Columbus, OH, United States

**Keywords:** cardioprotection, ischemic/reperfusion injury, cardiac remodeling, autophagy, HFpEF

Coronary heart disease (CHD) and its complications are the leading cause of death worldwide, even during the COVID-19 pandemic. A number of molecular pathologic pathways, including excessive reactive oxygen species, calcium overload, inflammatory responses, cardiac fibrosis, apoptosis, cell death, and mitochondrial dysfunction, etc., have been identified, which induce cardiovascular risk factors such as ventricles hypertrophy, tissue ischemia, arrhythmias and result in sudden cardiac death (SCD) and heart failure. The current Research Topic was designed to serve as a forum to publish research and review articles on new drugs, targets, and approaches to treating cardiac ischemic/reperfusion (I/R) injuries and preventing unfavorable heart remodelings such as hypertrophy and heart failure. Some of the studies published in this special issue are highlighted here.

The primary treatment of CHD consists of the recovery of coronary and other vessel revascularization, a phenomenon called reperfusion. However, reperfusion itself can generate reperfusion injury, leading to cardiomyocyte death and arrhythmias. The pathophysiological protective mechanism for I/R injury could be new vitamin D protective effects, presented by Lee et al. The investigation of the cells culture and animals with the use of hypoxia/reoxygenation (H/R) in a model study showed that the vitamin may protect mitochondrial structural and functional integrity and reduce mitophagy. More exactly it prevented H/R-induced apoptosis by inhibiting oxidative stress, modulating mitochondrial function, and inhibiting mitochondrial fusion and mitophagy by means of Drp1/Mff and BNIP3/LC3B pathways downregulation. According to the study byXiao et al., SUMOylation-mediate post-transcriptional modification plays a critical role in post-infarction cardiac remodeling. They found that the increased expression of E2 conjugation enzyme ubc9 induces an acceleration of autophagic flux, which can decrease cardiomyocyte apoptosis, reduce myocardial fibrosis and improve cardiac function post-MI. Ubc9 induces SUMOylation of the core proteins Vps34 and Beclin1 in PI3K-III, and then enhances the autophagic flux via augmenting the co-localization of Vps34 with autophagosome marker LC3 or lysosome marker Lamp1.

Cardiomyocyte hypertrophy is a common response of hearts to enhanced blood pressure or other stress. Autophagy has been shown to be involved in the development of myocardial hypertrophy. Zheng et al. further enrich the study of the molecular mechanism by which autophagy regulates cardiovascular diseases by demonstrating that Ang II induces cardiac hypertrophy by promoting excessive autophagy through SOCE/Orai 1. This study revealed the important role of SOCE/Orai1 in initiating hypertrophic growth of cardiomyocytes and extends our understating of the precise mechanisms of this pathologic phenomenon.

It is well known that heart failure with preserved ejection fraction (HFpEF) is responsible for half of heart failure cases. In a recent study, Zhang et al. used a high-salt diet-induced HFpEF rat model to study the potential protective role of Sacubitril/Valsartan Zhang et al. They found that intragastric administration of Sacubitril/Valsartan (68 mg/kg; i.g.) could protect rat hearts undergoing HFpEF. Further biochemical and histochemical studies revealed that TGF-β signaling is involved in Sacubitril/Valsartan mediated protection in HFpEF. This study provided new insights into the development of HFpEF and provides a potentially effective means of treating HFpEF.

More and more studies suggest that metabolic remodeling plays a critical role during the development of heart failure. Thus, targeting metabolic remodeling could serve as a novel therapy to treat heart failure. For this purpose, Li et al. tested the role of trimetazidine in protecting rat heart failure induced by subcutaneous injection of isoproterenol. As one of the most investigated drugs for energy metabolism, trimetazidine delivered orally showed protective effects, as evidenced by improving cardiac pump function, and reducing cardiac fibrosis and apoptosis. This study suggests the importance of energy metabolism during the development of heart failure.

The current special issue showcases several studies for novel targets and therapies to treat cardiac injuries and prevent heart failure. We hope this research will guide more basic, preclinical, and clinical studies, encouraging them to tackle the unmet medical needs of cardiac injuries.

